# A Public Health Minister

**Published:** 1903-10-31

**Authors:** 


### A PUBLIC HEALTH MINISTER.

Dr. F. Bushnell writes: Judging from analogy and in accordance with the trustworthy information that I have received from very varied sources there are many who sympathise with Surgeon-General George Evatt in his plea for a health minister for India in a recent number of the *British Medical Journal.*

Surgeon-General Evatt says, "at times one sees proposals put forward in an indefinite way for the appointment of a minister of health as a Cabinet Minister in our home English Government, to co-ordinate the work now done in health matters by the English, Irish, and Scotch Local Government Boards, the Home Office, the Army and Navy Medical Services, and the various smaller official organisations. No doubt amongst one of the first steps to be taken by the reformed British Medical Association will be a move in this direction."

As I have asked the opinion of the meetings of the Brussels Congress of Hygiene, the British Medical Association, the Sanitary Institute, and Royal Institute of Public Health, and many other associations and individuals on this important subject; and as I am, with many others, interested therein, I beg to say that the British Medical Association has it under serious consideration.

I believe that it is more important to create a popular vote in the interests of public health than to reform the War Office and reconsider even our fiscal relations. I suggest that there should be two steps taken: (*a*) the branches and members of the British Medical Association should associate themselves with members of the above-mentioned and kindred associations and with representatives of the governing bodies throughout the Empire, and eventually promote an inquiry by H.M. Government through the regular channels into the reconstitution of the Public Health State Department in accordance with present conditions. (*b*) That the subject should be raised above party or sect and even nations, and to this end the members, and especially the English members, of the international committees of future Congresses of hygiene and medicine should be asked to render the subject of health ministers "official" in the programmes. By this means the views of eminent men of all countries could be obtained, sound conclusions arrived at, and action taken.

There is no doubt that charity should begin at home, and I think that Mr. Balfour's words may be fairly applied to this matter, that being a "policy of humanity" it would be a "policy of wisdom."

There is, however, no need to labour the point, but it would be in accordance with our interests as well as our better conditions, to promote an Imperial Ministry of Health similar in one sense to that of Germany which might pave the way to a health minister for India. Hitherto the evolution of public health improvements has been consequent on panic, as witness the sanitary improvements that have followed outbreaks of cholera, small-pox, and plague. This is not common sense, and we need the steady and continuous educational "pressure" of a (Cabinet) Minister trained in the science of preventive medicine with responsible expert colleagues. The opinion of a statesman of such ripe experience as Lord Rosebery is that the relations of an expert head of a national department to the Cabinet could be settled by more methods than one.

